# Mindful Walking in Psychologically Distressed Individuals: A Randomized Controlled Trial

**DOI:** 10.1155/2013/489856

**Published:** 2013-07-31

**Authors:** M. Teut, E. J. Roesner, M. Ortiz, F. Reese, S. Binting, S. Roll, H. F. Fischer, A. Michalsen, S. N. Willich, B. Brinkhaus

**Affiliations:** ^1^Institute for Social Medicine, Epidemiology and Health Economics, Charité-Universtitätsmedizin Berlin, Luisenst ra**β**e 57, 10117 Berlin, Germany; ^2^Department of Psychosomatic Medicine, Charité-Universtitätsmedizin Berlin, 10117 Berlin, Germany; ^3^Immanuel Krankenhaus, Abteilung für Naturheilkunde, 14109 Berlin, Germany

## Abstract

*Background*. The aim of this randomized, controlled study was to investigate the effectiveness of a mindful walking program in patients with high levels of perceived psychological distress. *Methods*. Participants aged between 18 and 65 years with moderate to high levels of perceived psychological distress were randomized to 8 sessions of mindful walking in 4 weeks (each 40 minutes walking, 10 minutes mindful walking, 10 minutes discussion) or to no study intervention (waiting group). Primary outcome parameter was the difference to baseline on Cohen's Perceived Stress Scale (CPSS) after 4 weeks between intervention and control. *Results*. Seventy-four participants were randomized in the study; 36 (32 female, 52.3 ± 8.6 years) were allocated to the intervention and 38 (35 female, 49.5 ± 8.8 years) to the control group. Adjusted CPSS differences after 4 weeks were −8.8 [95% CI: −10.8; −6.8] (mean 24.2 [22.2; 26.2]) in the intervention group and −1.0 [−2.9; 0.9] (mean 32.0 [30.1; 33.9]) in the control group, resulting in a highly significant group difference (*P* < 0.001). *Conclusion*. Patients participating in a mindful walking program showed reduced psychological stress symptoms and improved quality of life compared to no study intervention. Further studies should include an active treatment group and a long-term follow-up.

## 1. Background

General psychological distress and stress-related diseases are considered to be an important health issue in Western societies. A recent survey with a representative sample showed that between 5% and 15% of the adult German population reported symptoms of marked psychological distress in the last 3 months, the frequency of chronic stress being higher among women than men [1, 2]. Another survey suggests that up to 80% of the German population feels distressed frequently, up to 30% most of the time [3]. It is also assumed that the share of workers who experience stress and psychological trouble due to their working conditions is increasing. In a recent German survey, one-fifth of 1756 employees felt overburdened on work; 43% reported that they experienced an increase of stress and work pressure in the last years [4]. Whereas a certain level of stress is generally considered to be of benefit to improve performance, continuous stress affects physical and mental health [5, 6].

In several studies, mindfulness training and physical exercise have demonstrated effects in reducing symptoms of psychological distress [7, 8]. Mindfulness training is a treatment strategy derived from Buddhist mindfulness meditation practice. It is described as the tendency to encounter moment-to-moment experiences without being lost in unhelpful or distressing thoughts triggered by the experience [9]. The most commonly studied mindfulness training program is mindfulness-based stress reduction (MBSR) that has demonstrated effects not only a wide range of mental and physical disorders, but also on stressed healthy people [7, 9]. A direct relationship between physical exercise and prevention or improvements of health has not only been established for many somatic diseases, but seems also plausible for psychological distress [8].

For the future, it is an important public health issue to develop and evaluate simple and cost-effective nonpharmacological therapies for the general population to prevent and treat individuals with acute or chronic psychological distress. 

Hypothetically it could be a good concept to combine mental and physical stress reduction strategies, especially if the combined exercise program would be easy to learn and run on low costs. Up to date, a combination between walking exercise and mindfulness has not been systematically evaluated. Therefore, we developed an easy to follow training program that combines mindfulness with walking. The aim of this randomized, controlled study was to investigate the effectiveness of mindful walking in patients with high levels of subjectively perceived psychological distress.

## 2. Methods

### 2.1. Design

This study was designed as an open, single-center randomized controlled trial including two parallel groups. All study participants gave their written informed consent before inclusion. The study was carried out at the outpatient clinic for Complementary and Alternative Medicine (CAM) at Charité Universitätsmedizin Berlin, Germany. The mindful walking training was performed in the surrounding streets and parks.

Patients were allocated to treatment groups by a randomization with a 1 : 1 ratio. The random allocation sequence was generated using SAS 9.2 software (SAS Institute Inc., Cary, NC, USA). After signing the informed consent form and completing the baseline assessment questionnaires, the subjects were centrally assigned to intervention or control group by an independent study nurse on the telephone line. Allocation was concealed according to the randomization list. 

The study was reviewed and approved by the Ethics Committee of the Charité Universitätsmedizin, Berlin, Germany (EA1/013/11 - 10.02.2011). The study was registered at ClinicalTrials.gov (NCT01716832).

### 2.2. Participants

Participants were recruited through advertisements in local daily newspapers. Study information and prescreening were undertaken by phone by a study nurse and a student of health science. Eligible subjects that reported a high level of psychological distress were invited for a personal consultation with a study physician for information, informed consent, and the assessment of inclusion or exclusion. Inclusion criteria were

- men and women between 18 and 65 years,
- increased level of psychological distress (visual analog scale >40 mm; range: 0–100 mm, higher values indicating more stress).

Exclusion criteria were

- regular walking training in the last 6 weeks (at least one regular training session per week),
- psychopharmacological drugs,
- regular mindfulness training (at least one regular training session per week),
- other CAM treatments against stress in the last 6 weeks,
- acute diseases or chronic disease at baseline,
- inability to walk.

The visual analogue scale for the detection of increased level of psychological distress was mainly chosen for practical reasons—VAS assessment is easily and quickly done.

### 2.3. Study Intervention

The intervention protocol was developed in a consensus process including a mindfulness-based stress reduction trainer, a sports therapist, and two medical doctors. Subjects allocated to the intervention group received 8 sessions of 60-minute mindful walking training within four weeks. Each training group consisted up to 15 individuals. The intervention was delivered by two sports therapists that were also trained in mindfulness-based stress reduction techniques. Each session was structured as follows:

- meeting at a defined meeting point and greetings (5 minutes);
- walking to a park (5 minutes);
- gymnastic exercises to warm up and short walking instructions (5 minutes);
- walking (10 minutes);
- mindful walking (10 minutes). Participants were instructed to mindfully observe and focus on their bodily sensations while walking remaining focused on their moment-to-moment experiences without being lost in unhelpful or distressing thoughts triggered by the experience. If this was experienced as a problem, the participants were instructed to focus their awareness on their breath while in- and exhaling,
- a feedback round was used to share and discuss the experiences (5 minutes),
- walking (10 minutes),
- gymnastic exercises (5 minutes),
- walking back to the meeting point (5 minutes).

Participants of the intervention group were encouraged and advised to keep on exercising for themselves after completing the 4-week program.

Participants allocated to the control group received no mindful walking training in the 12-week duration of the study (waiting list group). They were only sent study questionnaires after 4 and 12 weeks by postal service and had no consultations with the study physicians between baseline and week 12.

After the trial was completed, all subjects of the control group were offered the previously intervention in the above described manner for free.

### 2.4. Outcome Measures

Patients completed standardized questionnaires including outcomes at baseline and after 4 and 12 weeks. As a primary outcome measure, we defined the change to baseline of the score of Cohen's Perceived Stress Scale (CPSS) [10] after 4 weeks. The CPSS consists of 14 items including current levels of experienced and perceived stress. As secondary outcomes, we defined CPSS after 12 weeks, the subjective levels of psychological stress of the last week on a VAS (0–100 mm, higher scores indicate higher levels of stress) [11] and health-related quality of life (QoL) by the SF-36 questionnaire [12] (higher scores indicate higher QoL) after 4 and 12 weeks. Sociodemographic data of all participants was assessed at baseline. Adverse events were monitored by the mindful walking trainers throughout the study.

### 2.5. Statistical Analysis

The study was designed to detect a difference of the primary outcome parameter (difference between CPSS score to baseline) of 7 points between intervention and control group with a power of 90% including a dropout rate of approximately 20%. Therefore, we included 37 participants per group.

Data analysis is based on intention-to-treat population. Missing values were replaced by last observed value (last value carried forward). Primary and secondary outcome parameters were analyzed with analysis of covariance (ANCOVA), adjusted for the respective baseline value, two-sided with a significance level of 5%. The ANCOVA models were used to calculate adjusted differences with 95% confidence intervals. All statistical analyses were conducted in R 2.15.1 [13].

## 3. Results

**Figure 1:**
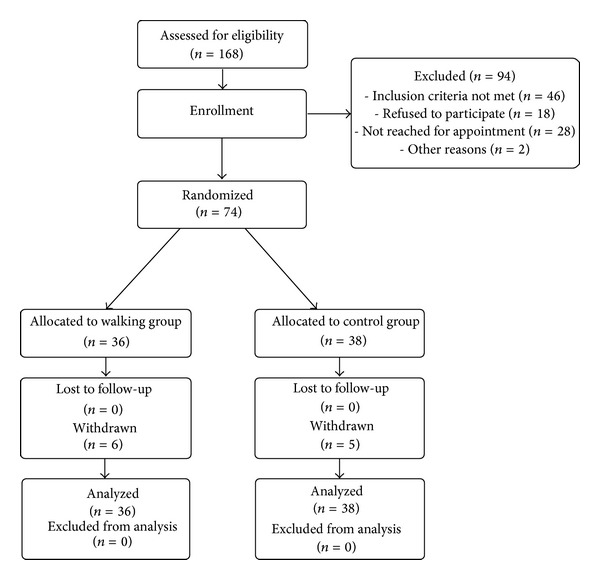
Trial flow chart.

Participants were included in the study from February to May 2011. Study intervention and followups were completed by September 2011. A total of 168 subjects were screened for eligibility; 94 could not be included. The main reason for noninclusion was not meeting the inclusion criteria mainly due to the subject reporting a perceived level of stress below 40 mm on the VAS (Figure 1). Seventy four patients were randomized with 36 allocated to the intervention, 38 to the control group. Five participants of the intervention group and 6 from the control group decided to terminate the study intervention program before their individual study end for several reasons: lost his house in a fire (*n* = 1), moving out of the city (*n* = 1), disease (*n* = 1), not explained (*n* = 2) in the intervention group and job-related stress (*n* = 1), not explained (*n* = 5) in the control group.

At baseline, the SF-36 Mental Component Score was significantly higher at baseline in the control group (36.3 ± 10.2) than in the intervention group (31.7 ± 8.8), all other characteristics showed comparable values (Table 1). The mean age of the participants was 52.3 ± 8.6 (SD) in the intervention and 49.5 ± 8.8 in the control group at baseline. Participants were in both groups predominantly women. The perceived stress intensity in the last week on the VAS can be considered as elevated at baseline in both groups (71.3 ± 13.1 mm in the study intervention and with 70.7 ± 12.4 in the control group). SF-36 Mental Component Score values in both groups were below the reported German average of the population whereas the Physical Component Score was comparable to the German average (both 50.0 ± 10.0) [12].

**Figure 2:**
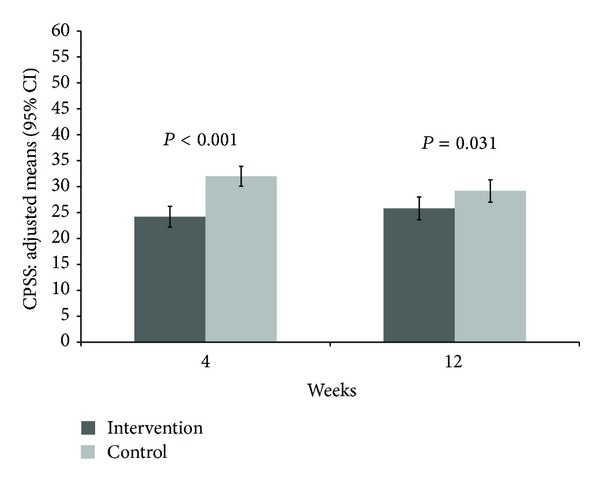
Adjusted means and 95% confidence intervals of Cohen's Perceived Stress Scale (CPSS) at 4 and 12 weeks with *P* values comparing mindful walking with no intervention (lower values indicating less psychological distress).

Adjusted CPSS differences to baseline after 4 weeks as primary outcome parameter were 8.8 [95% CI −10.8; −6.8] (mean 24.2 [22.2; 26.2]), in the intervention group and −1.0 [−2.9; 0.9] (mean 2.0 [30.1; 33.9]) in the control group, resulting in a statistically significant group difference (*P* < 0.001) (Table 2, Figure 2). Twelve weeks after baseline and 4 weeks after study intervention, CPSS remained still significant (*P* = 0.031) between study groups with a difference to baseline of −7.2 [−9.4; −5.0] in the intervention group and −3.8 [−6.0; −1.7] in the control group. 

**Figure 3:**
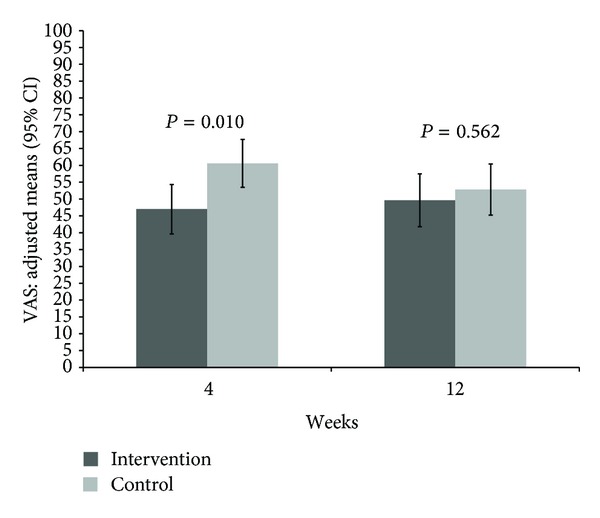
Adjusted means and 95% confidence intervals of perceived psychological distress on VAS (0–100 mm) at 4 and 12 weeks with *P* values comparing mindful walking with no intervention (lower values indicating less psychological distress).

Significant group differences were also found after 4 weeks for the VAS difference to baseline with −24.0 [−31.4; −16.7] (mean 47.0 [39.6; 54.3]) mm in the intervention group compared to −10.4 [−17.5; −3.3] (mean 60.6 [53.5; 67.7]) mm (*P* = 0.010) in the control group but not after 12 weeks (Table 2, Figure 3).

**Figure 4:**
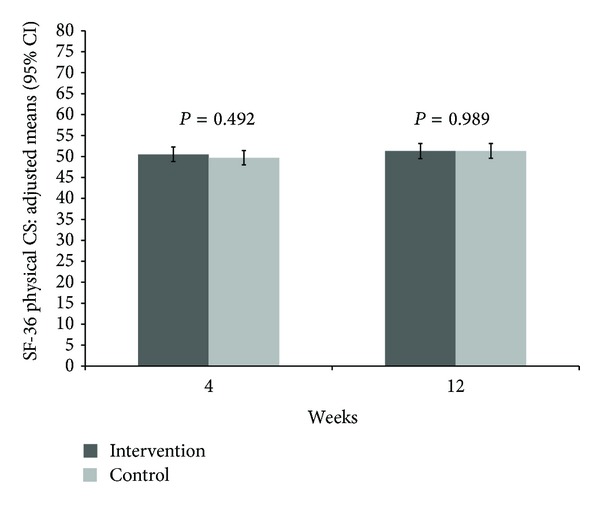
Adjusted means and 95% confidence intervals of SF-36 Physical Component Score at 4 and 12 weeks with *P* values comparing mindful walking with no intervention (higher values indicating higher QoL).

**Figure 5:**
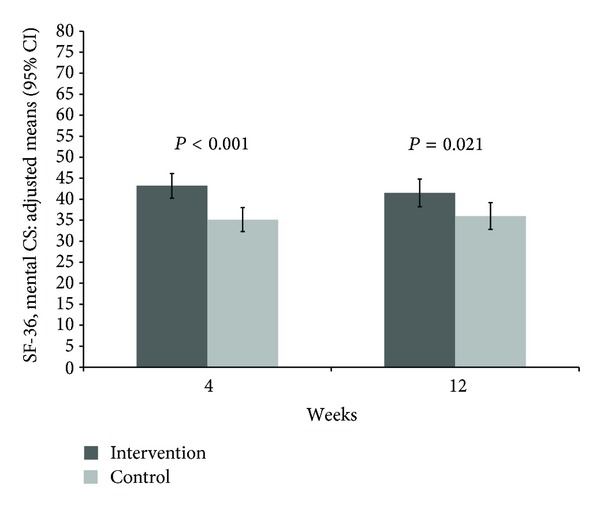
Adjusted means and 95% confidence intervals of SF-36 Mental Component Score at 4 and 12 weeks with *P* values comparing mindful walking with no intervention (higher values indicating higher QoL).

An improvement in the quality of life was observed for the SF-36 Mental Component Score after 4 weeks with a difference of 9.1 (6.2; 12.0) versus 1.1 (−1.8; 3.9) (*P* < 0.001) and 12 weeks with a difference of 7.5 (4.2; 10.8) versus 2.0 (−1.2; 5.2) (*P* = 0.021) but not for the Physical Component Score (Table 2, Figures 4, and 5). Significant group differences in favor of the study intervention were observed for the SF-36 scales mental health, vitality, emotional role function, and social role function after 4 weeks but only for the emotional role function after 12 weeks (Table 2).

Five participants of the intervention group reported that they kept on practicing the exercises regularly between 4 and 12 weeks whereas 14 participants reported noncontinuous or irregular practice. No serious adverse events were observed during the study. 

## 4. Discussion

In this study, statistically significant differences for CPSS, VAS, and SF-36 Mental Component Score were observed comparing mindful walking program to a no-treatment control group (waiting group) after 4 weeks. After 12 weeks from intervention start and 8 weeks after end of active treatment, the group differences were less marked, but CPSS and SF-36 Mental Component Score still showed a significant group difference. No serious adverse events were observed.

Mindful walking might be a useful new treatment strategy to reduce subjectively perceived symptoms of stress. In Germany, the normal charge for participation in an exercise class as this is 10 Euro per hour and participant (13 US$). The whole intervention costs 80 Euros (104 US$) per participant. Our study does not allow us to conclude if the combination of walking and mindfulness is superior to walking or mindfulness alone, but it definitely is a low-cost and easy to learn and implement exercise program.

Our data suggests that it is necessary to continuously practice mindful walking to obtain the best effects. However, only 5 participants of the intervention group kept on practicing regularly after the end of the intervention period although it was recommended to continue practicing. 

Strengths of this study include the use of highly experienced therapists, a confirmatory design, and a comparably high number of participants. 

However, there are some limitations which need to be discussed. A limitation of this study is the open design. Due to the nature of this trial, a blinding of the participants or study team was not possible. Also, this design makes it impossible to assess which components of the intervention were effective in reducing the stress. Possible effectors could have been exercise (walking), mindfulness practice, participating in a group, the expectation of improvement (9 of 10 subjects in both groups expected improvements) (Table 1), the suggestion that the program may reduce stress, or the received attention through study personnel. 

In addition, a design with a no-treatment control group imitates the situation of a patient who is asking himself: “Should I try this treatment or better do nothing and wait?” But this model does not answer the question about the best treatment or the most effective component. Another limitation is the low number of males participating. It is thus unclear whether the results of this trial are valid also for a male population. Generally a higher percentage of female participants do practice mind body therapies [14] and were also observed in an earlier study investigating the role of mindfulness in stress reduction [15]. 

To the best of our knowledge, this is the first randomized trial evaluating a mindful walking program for the treatment of perceived general psychological distress. There are not many stress reduction programs combining exercise with mindfulness practice or relaxation. Michalsen et al. [16] recently reported the results of a three-armed study where Iyengar yoga effectively reduced distress and improved related psychological and physical outcomes in seventy-two females. The practice of yoga consists of physical movements with isometric muscle strengthening, stretching, and flexibility, combined with a mental focus and an emphasis on mindfulness of body movements and consideration of breathing patterns. Other stress reduction programs showing positive results and combining exercise with mindfulness practice are Tai Chi [17] and Qi Gong [18, 19].

In this study, the exercise component consisted only of walking, which is easier to practice than yoga exercises, but misses the specific stretching and flexibility component. On the other hand, walking improves cardiovascular fitness and therefore might especially be valuable for individuals showing cardiovascular risk profiles.

Further studies about mindful walking in psychologically distressed individuals should compare the effects of the intervention with other active stress-reducing interventions. Comparing with a control arm delivering only guided walks (without a mindfulness component) and also a no-treatment control arm would allow to determine the effects of walking alone and additional mindfulness.

Another subject for research could evaluate gender-specific effects with the research question aimed to determine if mindful walking is also effective in men because the small percentage of men who participated in the trial allows no answer for this question.

## 5. Conclusion

Our results indicate that a 4-week mindful walking program might be a helpful tool to reduce subjectively perceived psychological distress compared to no intervention.

## Figures and Tables

**Table 1:** Baseline characteristics of participants in both study groups.

	Mindful walking intervention (*n* = 36)	No intervention control (*n* = 38)
Age (mean ± SD)	52.3 ± 8.6	49.5 ± 8.8
Gender (male, %)	4 (11.1%)	3 (7.9%)
Body mass index (mean ± SD)	24.6 ± 4.8	25.9 ± 4.6
Level of perceived psychological distress on visual analogue scale (mean ± SD)**	71.3 ± 13.1	70.7 ± 12.4
Cohen's Perceived Stress Scale (mean ± SD)**	33.1 ± 6.1	32.9 ± 7.0
Quality of life: SF-36—physical component score (mean ± SD)*	50.8 ± 8.3	50.1 ± 9.3
Quality of life: SF-36—mental component score (mean ± SD)*	31.7 ± 8.8	36.3 ± 10.2
Expectation of improvement (*n*, %)	32 (88.9%)	35 (92.1%)

*Higher values indicating better QoL, **lower values indicating less distress.

**Table 2:** Outcome measures at 4 weeks and 12 weeks (means and 95% confidence intervals adjusted for respective baseline value).

Outcomes	Adjusted differences to baseline (95% CI) mindful walking *n* = 36	Adjusted differences to baseline (95% CI) control (no intervention) *n* = 38	Adjusted means (95% CI) mindful walking *n* = 36	Adjusted means (95% CI) control (no intervention) *n* = 38	*P* value
*At 4 weeks *					
Cohen's Perceived Stress Scale (14 items)**	−8.8 (−10.8; −6.8)	−1.0 (−2.9; 0.9)	24.2 (22.2; 26.2)	32.0 (30.1; 33.9)	<0.001
Visual analogue scale (0–100 mm)**	−24.0 (−31.4; −16.7)	−10.4 (−17.5; −3.3)	47.0 (39.6; 54.3)	60.6 (53.5; 67.7)	0.010
SF-36—mental component score*	9.1 (6.2; 12.0)	1.1 (−1.8; 3.9)	43.2 (40.2; 46.1)	35.1 (32.3; 38.0)	<0.001
SF-36—physical component score*	0.1 (−1.7; 1.9)	−0.7 (−2.5; 1.0)	50.5 (48.8; 52.3)	49.7 (48.0; 51.4)	0.492
SF-36—vitality scale*	12.6 (7.7; 17.5)	2.9 (−1.9; 7.7)	50.2 (45.3; 55.1)	40.5 (35.7; 45.3)	0.006
SF-36—physical functioning scale*	0.6 (−2.37; 3.6)	−3.5 (−6.4; −0.6)	86.3 (83.3; 89.3)	82.2 (79.3; 85.1)	0.054
SF-36—bodily pain scale*	4.7 (−2.4; 11.9)	−1.3 (−8.2; 5.7)	73.2 (66.1; 80.3)	67.2 (60.3; 74.2)	0.238
SF-36—general health perceptions scale*	4.9 (0.4; 9.4)	−1.1 (−5.4; 3.3)	65.4 (60.9; 69.9)	59.4 (55.0; 63.8)	0.063
SF-36—physical role functioning scale*	11.3 (1.9; 20.8)	3.1 (−6.1; 12.3)	72.5 (63.0; 81.9)	64.2 (55.1; 73.4)	0.218
SF-36—emotional role functioning scale*	16.2 (4.3; 28.1)	−2.2 (−13.8; 9.4)	65.3 (53.4; 77.2)	46.9 (35.3; 58.5)	0.033
SF-36—social role functioning scale*	18.8 (12.2; 25.3)	4.9 (−1.5; 11.3)	74.7 (68.1; 81.2)	60.8 (54.5; 67.2)	0.004
SF-36—mental health scale*	13.3 (9.1; 17.5)	2.0 (−2.2; 6.1)	63.2 (58.9; 67.4)	51.8 (47.7; 56.0)	<0.001
*At 12 weeks *					
Cohen's Perceived Stress Scale**	−7.2 (−9.4; −5.0)	−3.8 (−5.7; −1.7)	25.8 (23.6; 28.0)	29.2 (27.0; 31.3)	0.031
Visual analogue scale**	−21.3 (−29.1; −13.5)	−18.2 (−25.7; −10.6)	49.6 (41.8; 57.5)	52.8 (45.2; 60.4)	0.562
SF-36—mental component score*	7.5 (4.2; 10.8)	2.0 (−1.2; 5.2)	41.5 (38.2; 44.8)	36.02 (32.8; 39.2)	0.021
SF-36—physical component score*	0.9 (−0.9; 2.7)	0.9 (−0.9; 2.7)	51.3 (49.5; 53.1)	51.3 (49.6; 53.1)	0.989
SF-36—vitality scale*	11.6 (6.5; 16.7)	6.4 (1.4; 11.3)	49.2 (44.1; 54.3)	43.9 (39.0; 48.9)	0.145
SF-36—physical functioning scale*	1.2 (−1.7; 4.1)	−1.3 (−4.1; 1.5)	86.7 (84.0; 89.7)	84.4 (81.6; 87.2)	0.226
SF-36—bodily pain scale*	4.7 (−1.7; 11.1)	3.2 (−3.0; 9.4)	73.2 (66.8; 79.6)	71.7 (65.5; 77.9)	0.742
SF-36—general health perceptions scale*	5.9 (1.0; 10.8)	0.4 (−4.4; 5.2)	66.4 (61.5; 71.3)	60.9 (56.1; 65.7)	0.119
SF-36—physical role functioning scale*	13.6 (5.0; 22.2)	9.5 (1.1; 17.8)	74.8 (66.2; 83.4)	70.6 (62.2; 79.0)	0.493
SF-36—emotional role functioning scale*	18.9 (7.4; 30.4)	0.5 (−10.7; 11.7)	68.0 (56.5; 79.5)	49.6 (38.4; 60.8)	0.027
SF-36—social role functioning scale*	14.2 (6.5; 22.0)	8.9 (1.3; 16.5)	70.2 (62.4; 77.9)	64.8 (57.2; 72.4)	0.336
SF-36—mental health scale*	9.2 (4.3; 14.1)	2.9 (−2.0; 7.7)	59.1 (54.1; 64.0)	52.8 (48.0; 57.6)	0.073

Abbreviations: *higher values indicating better QoL, **lower values indicating less distress.
